# Long-Term Development of Training Characteristics and Physiological Determinants in World-Leading and Medal-Winning Female Cross-Country Skiers: A Three Decade Comparative Analysis

**DOI:** 10.1186/s40798-025-00939-1

**Published:** 2025-11-21

**Authors:** Hanne Staff, Guro Strøm Solli, Boye Welde, Espen Tønnessen, Øyvind Sandbakk

**Affiliations:** 1 School of Sport Sciences, UiT The Arctic University of Norway, Tromsø, Norway; 2 Department of Sports Science and Physical Education, Nord University, Bodø, Norway; 3 School of Health Sciences, Kristiania University College, Oslo, Norway

## Abstract

**Background:**

This study describes the long-term development of training characteristics and physiological performance-determining variables among six world-class (Tier 5) female cross-country skiers across three decades, collectively holding 61 Olympic/World Championship medals.

**Methods:**

The skiers were divided into two groups: three world-leading (Tier 5+) athletes with ≥ 8 medals in international championships and three Tier 5 athletes with ≤ 4 medals. Self-reported training characteristics, as well as peak oxygen uptake (${\dot{\text{V}}}{\text{O}}_{{2{\text{peak}}}}$) and submaximal lactate threshold tests from junior to senior age were collected and analyzed retrospectively. Training was categorized into endurance, strength and speed training, with endurance training systemized using a five-zone intensity scale.

**Results:**

While athletes exhibited individual patterns in the development of training volume from junior to senior, the increase in training volume was primarily driven by a rise in low-intensity endurance training. There was a noticeable trend that Tier 5+ athletes had a longer, more consistent increase in training volume compared to Tier 5. Among Tier 5+ athletes, ${\dot{\text{V}}}{\text{O}}_{{2{\text{peak}}}}$ values showed a positive progression from junior to senior level, whereas only one of the Tier 5 showed the same pattern. Additionally, Tier 5+ demonstrated larger improvements in speeds at a given blood lactate and heart rate level than Tier 5 throughout the investigated period (e.g., mean increase in speed at estimated lactate threshold: ~14% for Tier 5+ vs. ~ 7% for Tier 5).

**Conclusions:**

Keeping in mind the limitations associated with the small sample size, the development pathways to becoming a Tier 5+ skier tended to involve longer, more consistent, increases in training volume, resulting in greater improvements in both ${\dot{\text{V}}}{\text{O}}_{{2{\text{peak}}}}$ and submaximal physiological determinants than Tier 5 skiers.

## Keypoints

- Despite individual development patterns, the increase in training volume from junior age to world-class senior level was primarily driven by increases in zone 1 low-intensity endurance training, with individual patterns in the progression of moderate- and high-intensity training.
- World-leading female cross-country skiers generally tended to demonstrate a longer, more gradual and stepwise increase in training volume, resulting in higher overall training volumes compared to their medal-winning peers.
- World-leading female skiers exhibited sustained development in ${\dot{\text{V}}}{\text{O}}_{{2{\text{peak}}}}$ and lactate threshold, surpassing medal-winning peers.

## Introduction

Cross-country (XC) skiing is an Olympic winter endurance sport in which training characteristics and physiological performance-determining factors have been extensively studied during the athlete’s peak performing years [1–6]. World-class XC skiers (i.e., Tier 5) are characterized by some of the highest peak oxygen uptake (${\dot{\text{V}}}{\text{O}}_{{2{\text{peak}}}}$_)_ values ever recorded [7], with ${\dot{\text{V}}}{\text{O}}_{{2{\text{peak}}}}$ values for female skiers typically ranging from 70 to 80 mL kg^−1^ min^−1^ [3–6, 8–10]. These ${\dot{\text{V}}}{\text{O}}_{{2{\text{peak}}}}$ values are 7–10% higher than those observed in national level female XC skiers, which also lead to lower physiological and perceptual strain at similar submaximal speeds [5].

A case study of a world-leading (i.e., multiple gold medal winning) female XC skier reported consistently high values of ${\dot{\text{V}}}{\text{O}}_{{2{\text{peak}}}}$ and ${\dot{\text{V}}}{\text{O}}_{{2}}$ at the lactate threshold (LT) during her five most successful seasons from the age of 30 to 35 [4]. However, this case study did not include data on the long-term development leading to these physiological values. A more recent study [11] examined this aspect and found no change of absolute ${\dot{\text{V}}}{\text{O}}_{{2{\text{peak}}}}$ values from junior to senior age among seven female Tier 5 athletes. Nonetheless, a significant 6.7% increase in body mass normalized (relative) ${\dot{\text{V}}}{\text{O}}_{{2{\text{peak}}}}$ was reported, rising from 66.9 ± 4.0 to 71.3 ± 4.4 mL kg^− 1^ min^−1^. The authors attributed the increase in relative ${\dot{\text{V}}}{\text{O}}_{{2{\text{peak}}}}$ to reduced fat mass caused by high training volumes. In a female world-class marathon runner, a 13 to 19% increase in speed at LT was reported from age 18 to 29, despite no change in relative ${\dot{\text{V}}}{\text{O}}_{{2{\text{peak}}}}$ [12]. These findings underscore the need for further research on the long-term development of physiological performance-determining variables, and their association with changes in training characteristics, among world-class endurance athletes [13].

The balance between training duration and intensity in the long-term development of endurance athletes remains a topic of debate [14, 15]. While world-class XC skiers train between 750 and 1050 annual training hours during their peak-performing senior seasons [1, 3, 4, 6], the long-term development in training volume from junior age to world-class level has been reported in only two previous studies. An 80% non-linear increase in annual training volume (from 522 to 940 h) was reported in a world-leading female XC skier [4], while a study on 17 Tier 5 XC skiers showed a mean increase of 203 ± 130 h in annual training volume [11], comparing only one junior and senior season, offering limited information about the continuous progression of training volume and intensity required to reach a world-class level.

In XC skiing endurance training accounts for more than 90% of the total training volume. Of this, ~ 90% is executed as low intensity training (LIT), with the remaining ~ 10% consisting of moderate—(MIT) and high-intensity training (HIT), primarily performed as competitions and interval sessions [1, 16]. How this training pattern develops during younger years remains unclear and warrant investigation. These observations highlight the need for detailed longitudinal data to better understand the progression of training characteristics associated with achieving world-class performance.

While current research has enhanced our understanding of the training characteristics distinguishing national from world-class athletes [5, 6], less is known about what separates athletes performing at the very highest level [4]. The Participation Classification Framework [7] published in 2022 has contributed to a more precise definition of athletes across different performance levels. However, the criteria for Tier 5 athletes, lack the sensitivity to distinguish world-leading athletes (i.e., athletes winning multiple international medals) from world-class athletes. Existing research suggests that the long-term development pathways of the world’s best endurance athletes are highly diverse [17–19], yet shared traits and unique features of world-leading athletes remain largely unexplored. To date, no studies have examined the long-term development of training and physiological determinants in world-leading versus world-class athletes. This highlights the need for comprehensive longitudinal research designs to identify and understand both differences and similarities.

Therefore, the current study aimed to describe similarities and differences in the long-term development of training characteristics and physiological performance-determining factors in world-leading and medal-winning female XC skiers across three decades.

## Methods

### Participants

Six Norwegian female world-class XC skiers (Tier 5) [7] were recruited for this study. The inclusion criteria consisted of (1) athletes who had achieved individual medals in the Olympic Winter Games (OWG) or World Championships (WCH), and (2) had systematically recorded their day-to-day training in detail from junior to senior world-class level and performed physiological testing at the Norwegian Olympic Training Center. The athletes were divided into two groups based on their competitive results: Tier 5+ includes athletes who have won 8 or more medals from OWG/WCH, and Tier 5 includes athletes who have won 4 or fewer medals. All athletes competed at the senior level between 1988 and 2023. The study was approved by the Norwegian Social Science Data Services (NSD Ref.nr. 995843), and all athletes gave their oral and written informed consent prior to study participation.

Performance data derived from the International Ski and Snowboard Federation (FIS) database [20] is presented in Table 1. In total the six athletes won 21 individual medals in OWG (10 gold medals) and 40 individual medals in the WCH (28 gold medals). The mean age at which athletes achieved their first podium performance at an international senior championship was 22.3 (19–24) years for Tier 5+ athletes and 24.7 (20–27) years for Tier 5 athletes (Table 1). The mean age at retirement was 33.7 (30–38) years for Tier 5+ athletes and 33.3 (32–35) years for Tier 5 athletes. Both the Tier 5+ and Tier 5 groups included two athletes who achieved a podium performance at the junior WCH and one athlete who achieved podium performance in the senior WCH while still being a junior. As seniors, the group of Tier 5+ athletes reached the podium in the WC, WCH, and OWG more than five times as often as the Tier 5 athletes.

**Table 1:** Overview of performance and career data for each skier included in the study

Level	Athlete	Time-period as senior athlete	Performance (Number of individual podiums in international competitions)				Analyzed years	Number of Lab tests^a^		Age at first senior podium performance	Age at retirement
			jr.WCH	WC	WCH	OWG		${\dot{\text{V}}}{\text{O}}_{{2{\text{peak}}}}$	LT		
Tier 5	a	1988–2003	0	20	0	2	18–29 year	15	13	27 year	35 year
	b	2007–2020	7	41	4	0	18–30 year	34	86	20 year	33 year
	c	2011–2023	2	10	0	1	18–30 year	21	49	24 year	32 year
Tier 5+	a^+^	1992–2003	1	60	5	3	18–30 year	37	28	24 year	30 year
	b^+^	2000–2018	0	168	17	10	19–33 year	29	64	23 year	38 year
	c^+^	2008–2022	3	128	14	5	18–27 year	29	50	19 year	33 year

jr. WCH, Junior World Championship; LT, Lactate threshold; OWG, Olympic Winter Games; ${\dot{\text{V}}}{\text{O}}_{{2{\text{peak}}}}$, Peak oxygen uptake; WC, World Cup; WCH, World Championship; yr., Year; ^a^Number of lab tests completed throughout the athlete’s career at the Norwegian Olympic Training Center

### Study Design

The study investigated the longitudinal development of training characteristics and physiological performance-determining factors from junior age (17–20 years) to senior world-class level. To cover both the transition from junior to senior levels (19–23 years) and the point of reaching world-class level, 10 to 15 seasons of training were analyzed for each athlete, covering the period from junior age to world-class level. The study aimed to compare Tier 5 athletes (a, b, c) with Tier 5+ athletes (a^+^, b^+^, c^+^), identifying similarities and differences in their development trajectories. To minimize the influence of time-dependent trends on training and performance, each athlete was paired with another athlete performing at the same time-period within the same decade (Table 1).

### Training Data

The athletes reported their training in either a web-based diary designed by the Norwegian Olympic Training Center, Microsoft Office Excel sheets, or written diaries designed by the Norwegian Ski Federation [1]. All three versions of the diary were based on the same intensity scale and organization of training data. The recorded training included information about frequency, duration, training forms (endurance, strength, speed) and intensity for endurance training. The registration of endurance, strength, and speed training is based on methods previously described [1, 5, 11, 21, 22]. All athletes used the five-zone intensity scale developed by the Norwegian Olympic Training Center [16]. For the data analysis, both the three- and the five-zone intensity scale were applied to enhance the clarity of illustrations (LIT = zone 1–2; MIT = zone 3; HIT = zone 4–5). This method of self-reported training, based on a modified session-goal approach, has previously been shown to provide valid and accurate measurement of training duration and intensity in XC skiers [21].

### Physiological Testing

The six athletes conducted a total of 455 (75.8 ± 30.6) physiological tests at the Norwegian Olympic Training Center. The athletes had different frequencies and consistency of laboratory tests. To provide a valid description of the long-term development of each athlete, only representative tests performed in a well-functioning state (i.e., no illness or injury), conducted in the general preparation period (August 1 st to November 30th), were included in the analyses. To illustrate the development from junior to world-class level, ${\dot{\text{V}}}{\text{O}}_{{2{\text{peak}}}}$ was presented as the highest value in the selected period for each season, while the development in the LT was presented with a representative test at the age of 19–20 years (junior age) and 27–33 years (world-class level) respectively.

The physiological tests registered in the database followed standard procedures at the Norwegian Olympic Training Center laboratory in Oslo, Norway. The testing procedure was typically conducted with the LT test first, followed by the ${\dot{\text{V}}}{\text{O}}_{{2{\text{peak}}}}$ test, separated by a rest period of a maximum of 5 min. In some cases, only the LT test was performed without a subsequent ${\dot{\text{V}}}{\text{O}}_{{2{\text{peak}}}}$ test, or only the ${\dot{\text{V}}}{\text{O}}_{{2{\text{peak}}}}$ test was conducted. Data from the laboratory indicate that performing an LT test, according to the described criteria, prior to the ${\dot{\text{V}}}{\text{O}}_{{2{\text{peak}}}}$ test does not affect the ability to reach ${\dot{\text{V}}}{\text{O}}_{{2{\text{peak}}}}$ in such highly trained individuals. The tests were performed while running on a treadmill. For the athletes included in the study, no systematic roller ski test results were available from their careers.

The protocol for LT tests included a 10-min standardized warm-up on the same treadmill used for testing. The test started at 6.6 km h⁻¹ with a 10.5% incline. Each stage lasted 5 min, followed by a 30-s recovery period. Treadmill speed increased by 0.9 km h⁻¹ at the start of each new stage. All tests were conducted on the same treadmill model (Woodway GmbH, 124 Weil am Rhein, Germany). Blood lactate concentration was measured using a YSI 1500 Sport Lactate Analyzer (YSI, Ohio, USA) from blood samples obtained from the fingertip immediately after each stage. Oxygen uptake was recorded between the third and fourth minute of each stage using an Oxycon Pro metabolic test system (Jaeger-Toennis, Würzburg, Germany) or the EOS Sprint System (Jaeger-Toennis, Würzburg, Germany, before 01.09.2004). Extensive testing was performed to ensure that the values measured with these two systems were comparable. Heart rate (HR) was measured during the last 30 s of each stage (Polar, Kempele, Finland). The estimated LT was defined by linear interpolation as ${\dot{\text{V}}}{\text{O}}_{{2}}$, HR, and speed at a blood lactate concentration 1.5 mmol L⁻¹ higher than the average blood lactate concentration based on the first two stages in the LT test. The number of stages varied between 4 and 7.

${\dot{\text{V}}}{\text{O}}_{{2{\text{peak}}}}$ was determined using an incremental protocol while running at a 10.5% incline. Detailed test criteria, procedures, and apparatus have been described previously [1, 10].

### Analyses

Because of the descriptive nature of the study as well as the low sample size in each group, only descriptive statistics (mean, standard deviation and range), are reported. Differences between athletes or seasons are presented in absolute numbers and percentages. Some missing data was identified in the training diaries, mainly for the transition period (March/April), in which discussions with the athletes helped fill the gaps. Missing training data that was not linked to injury or illness were reconstructed using documented years with complete records, validated through discussions with the athletes to ensure accuracy.

## Results

### Long-Term Development of Training

A total of 74 (12.3 ± 2.0) seasons were analyzed, comprising 34,650 (5775 ± 1181) sessions and 54,634 (9105 ± 2248) hours of training.

#### Training Volume and Training Content

**Fig. 1:**
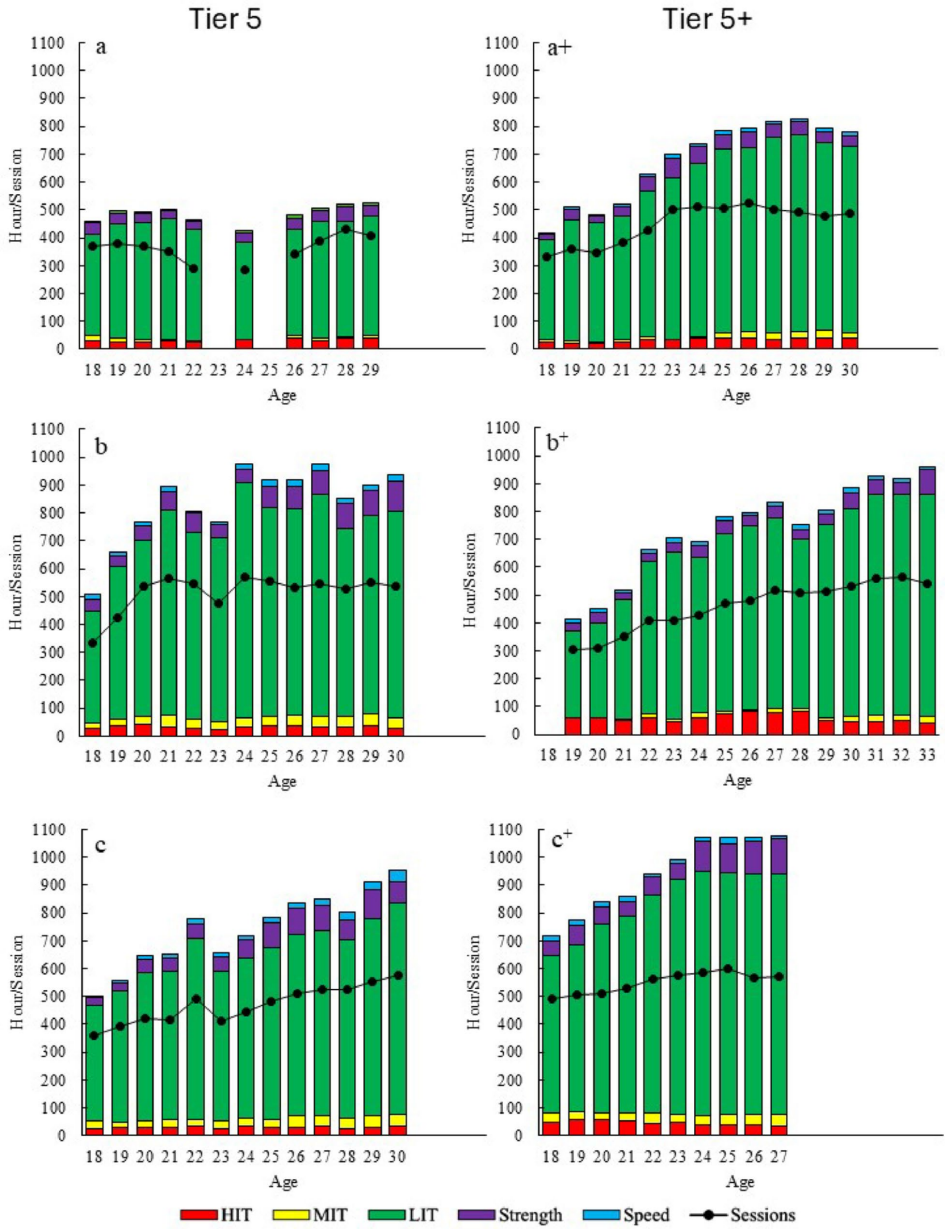
Annual progression of training characteristics for three Tier 5 and three Tier 5+ athletes [across three decades, see Table 1)], distributed into endurance [low-intensity (LIT), moderate intensity (MIT) and high-intensity (HIT)], strength, and speed training, along with the number of sessions

The annual progression of total training volume is presented in Fig. 1. While the athletes demonstrated individual patterns of development in both training volume and content, there was a general increase in training volume across decades. Aerobic endurance training accounted for 87–94%, strength training 5–11%, and speed training 1–3% of the total training volume throughout the investigated period. All athletes demonstrated a gradual increase in total training volume and the number of sessions, primarily explained by an increase in LIT. The annual training volume peaked between 525 and 1077 h, with a mean increase of 318 ± 168 h (60 ± 42%) from junior to senior world-class level (Fig. 1). Comparisons between the two groups revealed only minor differences in training volume during junior years, with Tier 5 averaging 572 ± 84 h (494–661 h) and Tier 5+ averaging 565 ± 187 h (412–774 h). There was a tendency towards a longer, more consistent and stepwise increase in training volume and the number of training sessions among Tier 5+ athletes, leading to higher peak annual training volumes (954 ± 126 h, ranging from 825 to 1077 h) compared to Tier 5 (818 ± 254 h, ranging from 526 to 976 h). However, significant inter-individual differences were observed: Athlete “a” consistently maintained a low training volume with minimal change throughout the studied period. In contrast, athlete c^+^ reported continued increases in her training volume during the following seasons (ages 27–33), but this training data was unavailable for inclusion in this study.

#### Aerobic Endurance Training

**Fig. 2:**
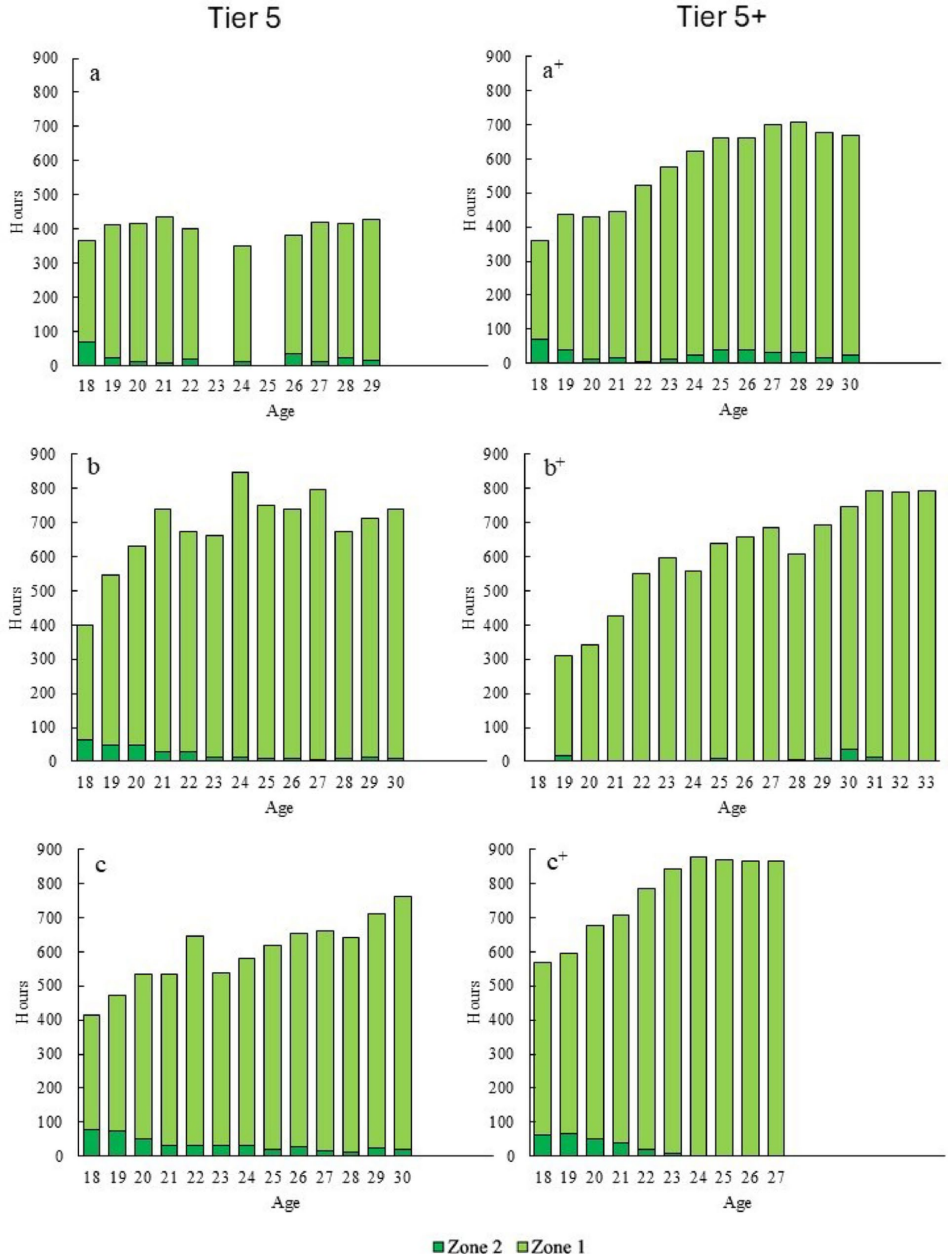
Annual progression of low-intensity training (LIT) for three Tier 5 and three Tier 5+ athletes (across three decades, see Table 1), distributed into zone 1 and 2 training

The distribution of LIT across intensity zones 1 and 2 is presented in Fig. 2. In total zone 1 accounted for 72–92% and zone 2 for 0–18% of the total LIT volume across seasons and athletes. The gradual increase in LIT was primarily caused by an increase in zone 1, while most athletes show a reduction in the volume of zone 2 training from junior to senior levels.

**Fig. 3:**
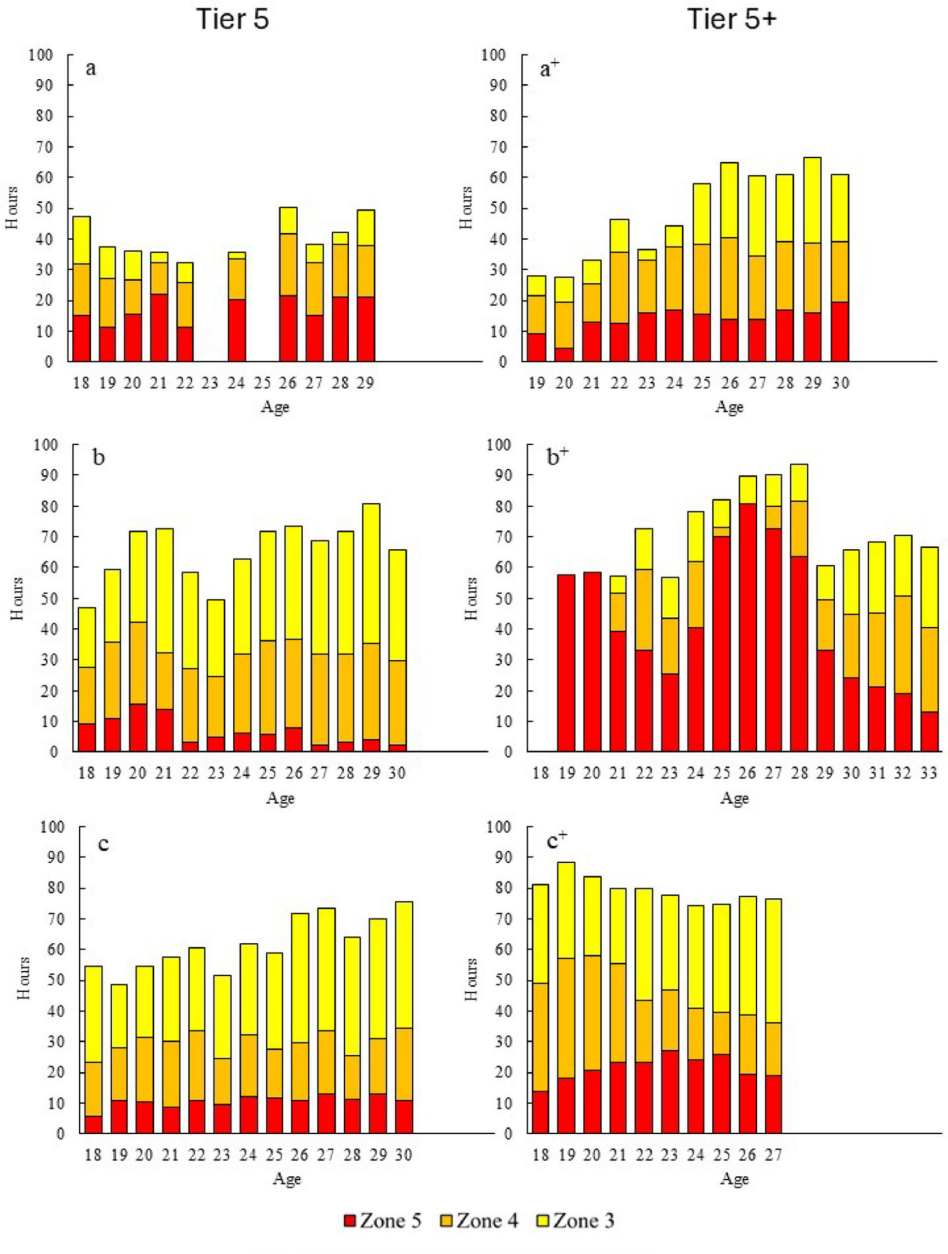
Annual progression of moderate- (MIT; including zone 3) and high-intensity training (HIT; including zone 4 and 5 training) for three Tier 5 and three Tier 5+ athletes (across three decades, see Table 1)

The annual progression of MIT and HIT across intensity zones 3, 4, and 5 is presented in Fig. 3. The total annual volume of MIT and HIT ranged from 50 to 90 h across seasons and athletes. The athletes exhibited individual patterns in the progression of MIT and HIT without any distinct trends within or between groups. Athletes a^+^ and b^+^ both showed increases in total MIT/HIT volume from junior to senior levels but differed in both the distribution and allocation of time across intensity zones 3–5. The remaining athletes demonstrated fluctuating patterns between seasons, with a general tendency towards increased time spent in zone 3 vs. zone 4 and 5 from junior to senior levels.

#### Strength and Speed Training

**Fig. 4:**
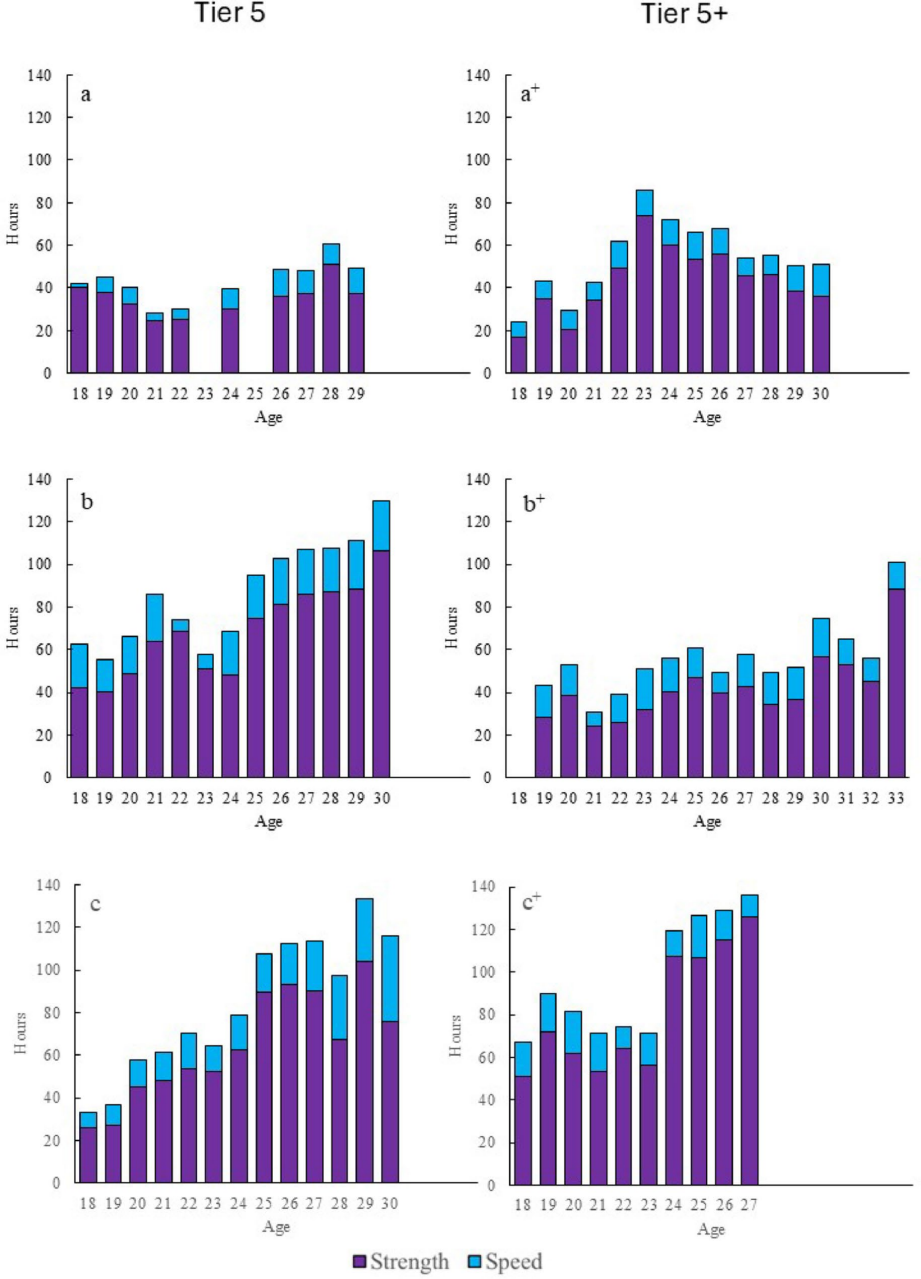
Annual progression of strength and speed training for three Tier 5 and three Tier 5+ athletes (across three decades, see Table 1)

The annual progression of strength and speed training is presented in Fig. 4. The development of strength training was relatively similar across all athletes at junior age, but distinct differences in development were observed throughout the senior age (Fig. 4). No systematic differences were detected between Tier 5 and Tier 5+; rather, our analysis suggest that the observed differences are more likely related to periods in which strength training was given greater emphasis. Athlete a, a^+^ and b^+^ showed fluctuations or small increases during the senior age before stabilizing around 40–60 h per year. In contrast, athlete b, c and c^+^ exhibited a rapid increase in time spent on strength and speed training, escalating from approximately 40–60 h to 100–140 h annually by the age of 24–30 years.

The volume of speed training was relatively stable across the investigated seasons. However the athletes who increased their strength training volume also tended to allocate more time to speed training.

### Long-Term Development of Peak Oxygen Uptake and Lactate Threshold

The six athletes completed a total of 165 (27.5 ± 8.2) ${\dot{\text{V}}}{\text{O}}_{{2{\text{peak}}}}$ tests and 290 (48.3 ± 25.8) LT tests at the Norwegian Olympic Training Center. The number of tests conducted varied both among athletes and across seasons within each athlete (Table 1).

#### Peak Oxygen Uptake

**Fig. 5:**
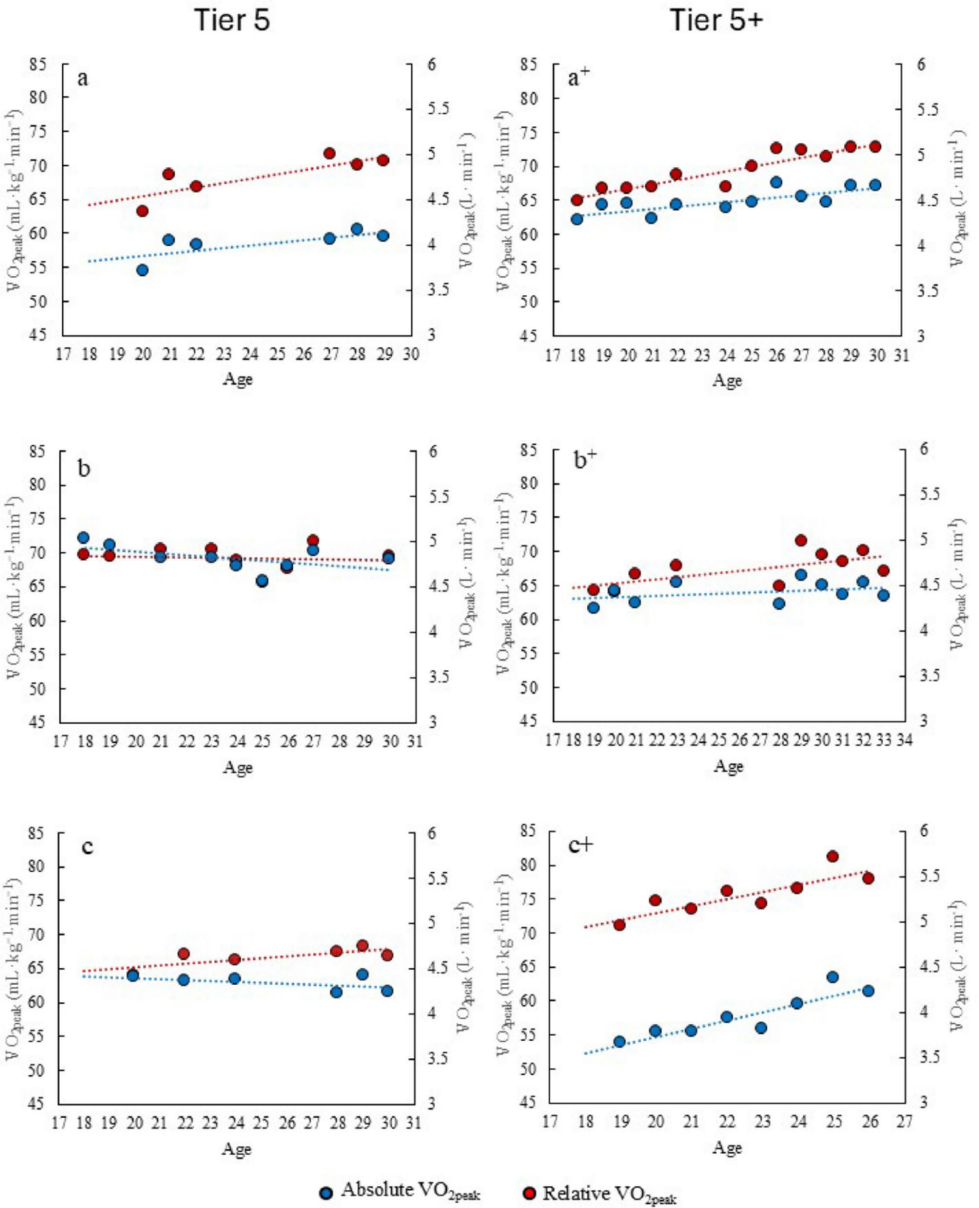
The long-term development of absolute (blue, additional y-axis) and relative (red) ${\dot{\text{V}}}{\text{O}}_{{2{\text{peak}}}}$ values for three Tier and three Tier 5+ athletes (across three decades, see Table 1)

The development of ${\dot{\text{V}}}{\text{O}}_{{2{\text{peak}}}}$ from junior to senior world-class level is presented in Fig. 5.

At the junior age the mean relative ${\dot{\text{V}}}{\text{O}}_{{2{\text{peak}}}}$ were 66.4 ± 3.3 mL kg^−1^ min^−1^ (range: 63–74 mL kg^−1^ min^−1^) peaking at 72.3 ± 4.6 mL kg^−1^ min^−1^ (range: 68–81 mL kg^−1^ min^−1^) at senior age. In absolute terms, ${\dot{\text{V}}}{\text{O}}_{{2{\text{peak}}}}$ values averaged 4.3 ± 0.5 L min^−1^ (range: 3.7–5.0 L min^−1^) at junior age and peaked at 4.5 ± 0.2 L min^−1^ (range: 4.2–4.8 L min^−1^) at senior age. All athletes demonstrated an increase in relative ${\dot{\text{V}}}{\text{O}}_{{2{\text{peak}}}}$_,_ from their best test as juniors to their highest level at senior age (+ 5.9 ± 2.7 mL kg^−1^ min^−1^, range: 2.0–11.1 mL kg^−1^ min^−1^). However, improvements in absolute ${\dot{\text{V}}}{\text{O}}_{{2{\text{peak}}}}$ were less consistent (+ 0.2 ± 0.3 L min^−1^, range: −0.1–0.6 L min^−1^), with one athlete reaching her highest absolute ${\dot{\text{V}}}{\text{O}}_{{2{\text{peak}}}}$ at the age of 18. Tier 5+ generally displayed a greater percentage increase in ${\dot{\text{V}}}{\text{O}}_{{2{\text{peak}}}}$ values than Tier 5 athletes. For absolute values, Tier 5 athletes improved by 3.3 ± 7.8%, while Tier 5+ improved by 6.0 ± 8.3%. For relative values Tier 5 athletes improved by 6.7 ± 3.9% and Tier 5+ by 11.0 ± 2.8%.

The athletes´ best ${\dot{\text{V}}}{\text{O}}_{{2{\text{peak}}}}$ test performed in the second half of the general preparation (August to November) across seasons is presented in Fig. 5. Tier 5+ athletes demonstrate a clearer trend of gradual, positive development over the years. This pattern is only observed in one of the Tier 5 athletes (a), while the other Tier 5 athletes show fluctuations around similar values throughout their careers.

#### Lactate Threshold

**Fig. 6:**
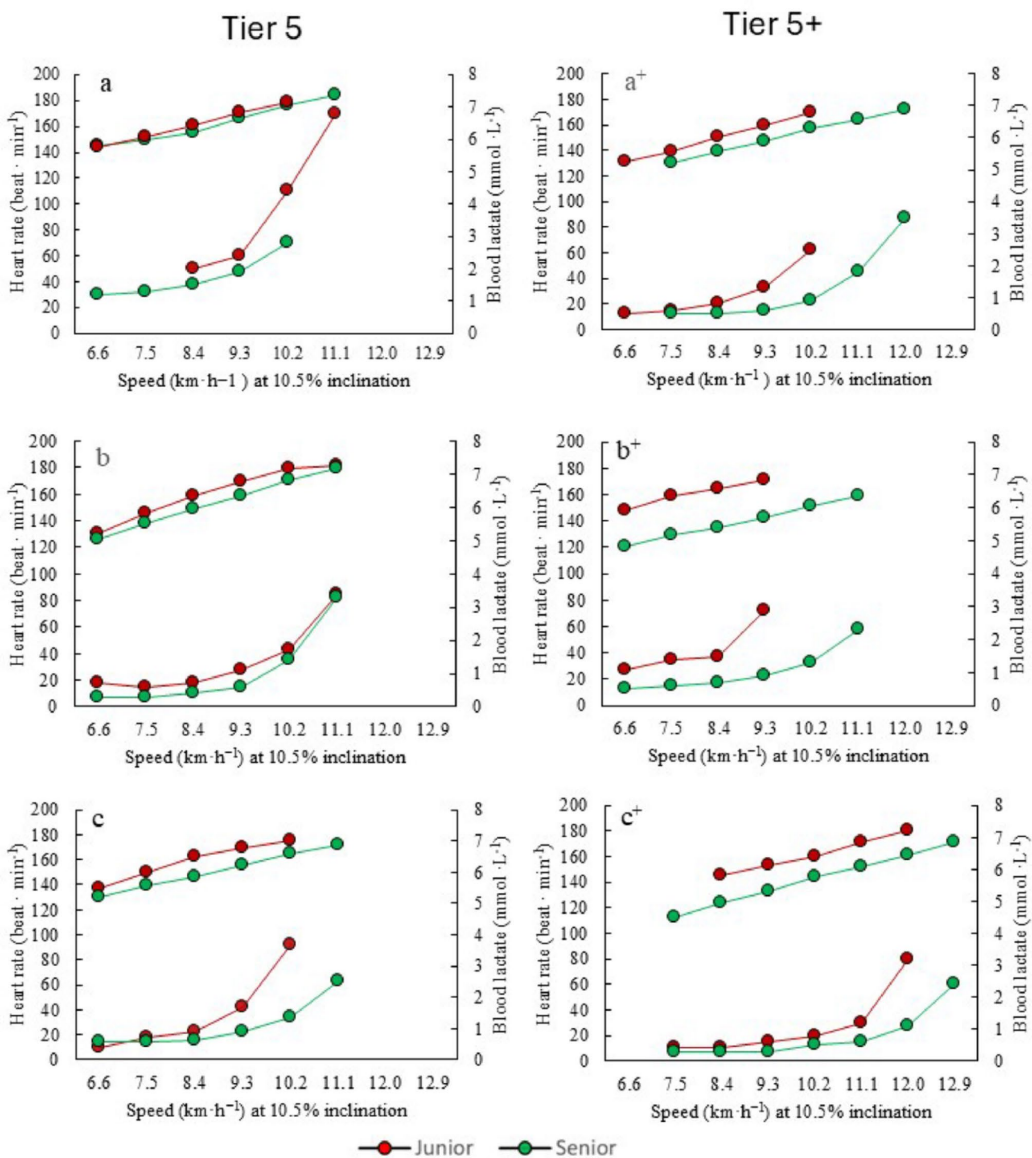
Development of lactate threshold tests from junior (red) to senior world-class level (green) for three Tier 5 and three Tier 5+ athletes (across three decades, see Table 1). Heart rate plotted against speed is presented at the upper lines (y-axis) and blood lactate values presented at the lower lines (secondary y-axis)

The development of HR and blood lactate concentration measured during the LT tests and plotted against speed, from junior to senior world-class level, is presented in Fig. 6.

The athletes generally exhibited a rightward shift (i.e., consistently higher speed at equivalent heart rate or blood lactate concentration) along with a down regulation (i.e., lower lactate levels at the first 2–3 steps of the LT test). Notably, all Tier 5+ athletes demonstrate a more pronounced right shift in their lactate profile compared to Tier 5 athletes. Additionally, all athletes, except athlete a, show a lower HR at given speeds during senior compared to junior age.

**Fig. 7:**
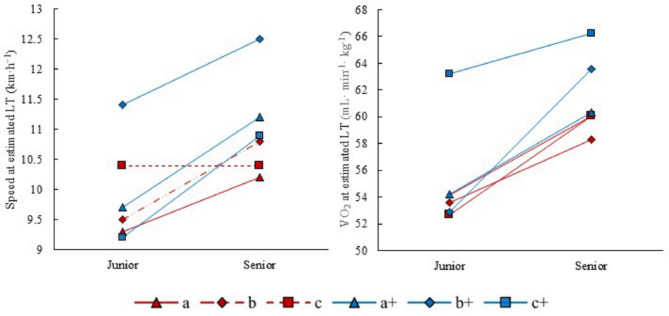
Development of speed (running at 10.5% inclination) (**A**) and oxygen uptake (${\dot{\text{V}}}{\text{O}}_{{2}}$) (**B**) at the estimated lactate threshold (LT) from junior to senior world-class level for three Tier 5 (red) and three Tier 5+ (blue) athletes (across three decades, see Table 1)

Speed and ${\dot{\text{V}}}{\text{O}}_{{2}}$ at the estimated LT during a junior and senior world-class season are presented in Fig. 7A-B. There are large individual differences in the speed at which LT occurs. For all athletes included the average speed at the estimated LT was 9.9 ± 0.8 km h^−1^ (range: 9.2–11.4 km h^−1^) at junior age, increasing to 11.0 ± 0.8 km h^−1^ (range: 10.2–12.5 km h^−1^) at senior age. The mean VO_2_ at LT during junior age was 55.1 ± 4.0 mL kg^−1^ min^−1^ (range: 58.3–63.22 mL kg^−1^ min^−1^), increasing to 61.4 ± 2.9 mL kg^−1^ min^−1^ (range: 52.7–66.2 mL kg^−1^ min^−1^) at senior age. Overall, Tier 5+ athletes exhibited a greater increase in LT speed from junior to senior age compared to Tier 5 (14% vs. 7%), while the increase in ${\dot{\text{V}}}{\text{O}}_{{2}}$ at the estimated LT was approximately similar across groups (~ 11%).

## Discussion

This study describes similarities and differences in the long-term development of training characteristics and physiological performance-determining factors in world-leading (Tier 5+) and medal-winning (Tier 5) female XC skiers across three decades. The main findings were as follows: (1) although individual development patterns varied, training volume consistently increased from junior to world-class level in both groups, predominantly driven by increased zone 1 LIT. (2) While, no clear differences in training volume were observed between groups during junior age, Tier 5+ athletes demonstrated a longer, more gradual and stepwise increase in training volume, generally leading to higher peak training volume compared to Tier 5 athletes. (3) No clear pattern in intensity distribution differences was observed within or between groups. (4) Tier 5+ athletes had a more consistent development of both maximal and submaximal performance-determining variables compared to Tier 5.

### Long-Term Development of Training Characteristics

#### Training Volume and Training Content

The peak training volume (525–1077 h) reported in this study is, on average, consistent with previous reports on world-class XC skiers [1, 4–6]. However, the range is larger than previously documented, with the lowest value notably below what has earlier been reported among world-class skiers. As shown in prior studies, endurance training accounted for ~ 90% of the total training volume [23]. One athlete competing in the earliest period completed a relatively low training volume (~ 500 h), yet performed at a world-class level, whereas some of the more recent athletes nearly doubled this training volume. The professionalization of elite sport over recent decades has likely contributed to increased training volume among female athletes [24, 25], and our data also indicate that modern XC skiers are reaching higher training volumes at younger ages compared with earlier generations.

Although large individual differences in the progression of training volume were observed, Tier 5+ athletes demonstrated a gradual, stepwise increase in training volume over a longer period compared to less consistent progression observed in Tier 5 athletes in the transition from junior to senior world-class level. The increase in training volume was primarily driven by an increase in the number of sessions, while session duration only increased modestly. Training volume during junior age did not appear to be a decisive factor for achieving Tier 5 vs. Tier 5+ performance at the senior level. However, the more consistent progression in training volume allowed Tier 5+ to achieve greater overall increase in training volume from junior age to senior world-class level. On average, Tier 5+ athletes increased their training volume by nearly 70% compared with 43% among Tier 5 athletes, and this exceeds the 35% increase previously reported in female world-class XC skiers over ~ 8 years [11]. Overall, these results indicate that Tier 5+ athletes accumulated higher training volumes, which aligned with their superior performance level and were partly explained by greater continuity in training. To better understand the underlying factors contributing to achieve continuity, future studies should conduct long-term prospective tracking of female XC skiers as they progress from junior to senior levels, including systematic monitoring of training characteristics, performance, illness, injury, and “life balance factors” (e.g., school, studies, work).

#### Aerobic Endurance Training

In line with previous studies, the increase in training volume was primarily driven by increased volume of zone 1 LIT [4, 11, 26]. This appears rational, as the quality of LIT training is likely higher both from a physiological and technical perspective at older ages, when work economy and speed within this zone are more fully developed [12]. Moreover, since the investigated athletes already as juniors had HIT volumes corresponding to previously reported training data for world-class skiers, it seems reasonable that further progression in training load is primarily achieved through increases in LIT (and MIT) [11].

Zone 2 LIT training was more common during junior years compared to senior age. One possible explanation is the lower speed in zone 1 at the junior level, which may cause junior athletes to reach zone 2 more easily particularly when skiing across hilly terrain. Since maintaining proper skiing technique requires a minimum speed, this could naturally result in more zone 2 training among junior athletes [27–29]. In addition, the lower total training volume at the junior stage likely allows for more zone 2 training without generating excessive load, in contrast to world-class athletes who operate at much higher overall training volume [29].

The total annual volume of MIT and HIT, ranging from 50 to 80 h, aligns with previous findings at both junior and senior levels. No distinct patterns emerged in the development of MIT or HIT within or between groups, although there was a general tendency for increased MIT (zone 3) volumes from junior to senior levels. Similar trends have been reported in other studies [4, 30], possibly linked to the rightward shift of the lactate profile, allowing athletes to perform MIT at higher speeds.

The development of HIT volume was inconsistent across athletes from junior to senior level. For example, one athlete performed relatively high HIT volumes (70–80 h) during some senior seasons due to adoption of HIT block periodization regimes that gained popularity among Norwegian endurance athletes in the early 2000 s [14, 31]. While block periodization of HIT may lead to rapid performance gains [32], the effectiveness on long-term development of this periodization strategy is uncertain [14]. Other athletes showed a more consistent pattern, and we found no evidence that higher HIT training volume led to greater development or higher performance in our data.

#### Strength and Speed Training

The volume of strength and speed training was relatively similar among athletes during their junior years. However, large differences emerged in the development of strength training throughout the senior years.

Athletes from more recent decades demonstrated a steady increase in total strength training from the age of 24–25 (60–80 h per year), eventually plateauing at 100–120 h per year. This trend likely reflects evolving knowledge and growing emphasis on the role of strength training in XC skiing [23, 33, 34]. The increased focus on strength training may be attributed to its importance for technique development at higher speeds, as well as the demands of newer competition formats such as mass start and sprint events. Greater attention to optimizing training for female athletes may also have contributed to this trend [24]. Supporting this, Walther et al. [11] observed an increase in strength training from junior to senior age for female athletes, but not for males, in their study of world-class XC skiers. This aligns with a previous study, reporting larger strength training volume in female than male XC skiers and biathletes [35]. It has been suggested that female skiers may have greater potential to improve upper-body strength and endurance than their male counterparts, likely due to lower baseline upper-body muscle mass [36]. However, it remains unclear whether female athletes require greater overall strength training compared to male athletes [37]. Future studies should investigate the long-term development of strength training and its transferability to XC skiing performance, including both male and female athletes and examining the effects of targeted upper-, core- and lower-body muscle groups [33].

While strength training is typically executed in the gym, the development of speed is closely linked to the specificity of movement patterns and is therefore mainly carried out as roller skiing or XC skiing [38]. Interestingly, the athletes with the highest strength volumes also engaged in more speed training. This connection highlights the interplay between strength and speed capacities and likely their role in the development of technique [39, 40]. Future and ongoing research on female athletes will hopefully contribute to the understanding of optimizing strength and speed training for female XC skiers.

### Development of Peak Oxygen Uptake and Lactate Threshold

#### Peak Oxygen Uptake

World-class XC skiers have shown some of the highest ${\dot{\text{V}}}{\text{O}}_{{2{\text{peak}}}}$ values, with values of 80–90 and 70–80 mL kg^−1^ min^−1^ for men and women, respectively [3–6, 8–10]. This is confirmed in the present study, where we also publish one of the highest ever reported values of a female XC skier with 81.1 mL kg^−1^ min^−1^, with repeated test values exceeding 79 mL kg^−1^ min^−1^ during her senior career. Additionally, this study provides unique data on the long-term development of ${\dot{\text{V}}}{\text{O}}_{{2{\text{peak}}}}$ over 10–15 years. Tier 5+ athletes showed a gradual positive development of both absolute and relative ${\dot{\text{V}}}{\text{O}}_{{2{\text{peak}}}}$ values from junior to senior world-class levels, whereas no clear pattern was observed for Tier 5 skiers. In contrast Walther et al. [11], only reported increases in relative ${\dot{\text{V}}}{\text{O}}_{{2{\text{peak}}}}$ values from junior to senior levels in female Tier 5 athletes, explained by a slight decrease in body mass. The present study therefore indicates an increase in absolute ${\dot{\text{V}}}{\text{O}}_{{2{\text{peak}}}}$ among Tier 5+, which was not observed in Tier 5 athletes or reported in previous research. In summary, these findings challenge the statements that ${\dot{\text{V}}}{\text{O}}_{{2{\text{peak}}}}$ reaches its full potential early in an athlete’s development [12] with limited possibility for further development. Interestingly the data from this study shows small but consistent increases in ${\dot{\text{V}}}{\text{O}}_{{2{\text{peak}}}}$ also after the age of 24–25 in Tier 5+ athletes. Overall, this study indicates that ${\dot{\text{V}}}{\text{O}}_{{2{\text{peak}}}}$ can continue to improve through individualized progression in training, at least in world-leading athletes.

#### Lactate Threshold

There is generally a lack of studies examining the long-term development of submaximal performance-determining variables in endurance sports, including XC skiing [13]. The suggestion that these variables improve consistently over time across endurance sports, in contrast to more modest improvements typically observed in ${\dot{\text{V}}}{\text{O}}_{{2{\text{peak}}}}$ [13], was confirmed by our findings. The more positive development of ${\dot{\text{V}}}{\text{O}}_{{2{\text{peak}}}}$ values from junior to senior levels among Tier 5+ than Tier 5 athletes, was accompanied by more distinct improvements in submaximal performance indicators. The LT tests clearly revealed a more pronounced rightward shift in the lactate curve and higher speeds at the estimated LT in Tier 5+ athletes, although all athletes showed positive development. While, causative analysis was not conducted, these larger improvements in Tier 5+ athletes could be linked to their higher training continuity and more gradual progression in training volume [4, 12].

Interestingly, our data indicate a shift from ${\dot{\text{V}}}{\text{O}}_{{2{\text{peak}}}}$ testing as the most used laboratory assessment to a greater emphasis on submaximal LT tests during the recent decades. This underscores the LT test as a valuable tool for athletes and coaches in the evaluation of training load and adaptations. A strength of the test is that it easily can be integrated into the athletes daily training [41]. However, ${\dot{\text{V}}}{\text{O}}_{{2{\text{peak}}}}$ testing remains essential for understanding the physiological profile of the athletes and assessing the most important prerequisites for further performance development.

In this study the physiological tests analyzed were conducted as running during the general preparation phase. In the future, collecting more data on physiological variables measured in sport-specific exercise at multiple timepoints throughout the season for world-leading athletes of both sexes, would be beneficial for understanding how changes in training and performance-determinants evolve [42].

### Strengths and Limitations

It should be acknowledged that innate genetic dispositions and other factors beyond the scope of this study, may have played a decisive role in determining performance differences between Tier 5+ and Tier 5 athletes. Qualitative in-depth interviews could have provided valuable insight into additional factors contributing to their development. Another limitation is the variability in the precision of training data logging among athletes, which necessitates interpreting results in terms of general trends and patterns rather than absolute values. Furthermore, the laboratory tests were conducted using uphill treadmill running rather than sport-specific modes such as roller skiing or XC skiing. Still, uphill running remains a valid indication of general aerobic endurance capacity since it is less influenced by technique.

## Conclusion

This study describes the unique development pathways of six female world-class XC skiers, providing benchmark values in training characteristics and physiological profiles associated with reaching the highest performance level. The findings are particularly relevant for coaches and athletes in endurance sports, emphasizing the importance of continuity, gradual progression, and a high degree of individualization throughout the development process.

Despite individual differences, the increase in training volume from junior age to the world-class level was primarily driven by increases in zone 1 LIT, with a tendency for increased zone 3 MIT training. No consistent patterns in the progression of speed or strength were observed. However, Tier 5+ athletes tended to have longer periods of gradual, stepwise increases in training volume compared to Tier 5, which was associated with greater improvements in both maximal and submaximal physiological determinants. Tier 5+ athletes demonstrated a gradual development of ${\dot{\text{V}}}{\text{O}}_{{2{\text{peak}}}}$ from junior to senior age and beyond the age of 24–25, whereas no clear pattern was evident for Tier 5. In both groups, the most distinct improvements were higher speeds at a given blood lactate and HR, indicating enhanced LT, with Tier 5+ athletes generally showing more pronounced gains.

Importantly, a trend toward increased total training volume across decades was observed, likely reflecting increased knowledge and professionalization in XC skiing. However, junior training volume did not predict senior-level training volume or performance, supporting the need for individual progression rather than fixed benchmarks. Furthermore, both maximal and submaximal performance indicators continued to develop beyond junior age, underscoring the importance of keeping female athletes in structured development programs for a longer period and tailoring training strategies to each athlete’s starting point and evolving needs.

Overall, the unique development pathways of world-leading athletes are characterized by gradual, stepwise increases in training volume, resulting in higher accumulated loads and superior physiological development compared with their lower-performing peers.

## Data Availability

The datasets used and/or analyzed during the current study are available from the corresponding author upon reasonable request.
